# Queries regarding the two papers each reporting on 1000 essential thrombocythemia patients in BCJ

**DOI:** 10.1038/s41408-024-01063-1

**Published:** 2024-05-31

**Authors:** Martin H. Ellis

**Affiliations:** 1 Hematology Institute and Blood Bank, Meir Medical Center, Kfar Saba, Israel; 2 School of Medicine, Faculty of Medicine and Health Sciences, Tel Aviv University, Tel Aviv, Israel

**Keywords:** Myeloproliferative disease, Myeloproliferative disease

I read with interest the latest significant contributions of the Florence [1] and Mayo Clinic [2] myeloproliferative neoplasm groups to our understanding of essential thrombocytosis (ET). Their contribution of 1000 patients each, fully annotated and followed for decades, provides unique insights into the disease. I would appreciate additional information and the authors’ thoughts regarding the following questions relating to the triple negative (TN) patients in the cohorts:

First, was there a group of TN patients who were also negative for all passenger mutations ie who had no clonal marker at all? If so, did they have a different clinical and hematologic phenotype and/or bone marrow histologic findings to the remainder of the TN patients who did have a clonal marker?

Second, the TN patients in the Florence cohort seem to have a far better prognosis regarding myelofibrosis, leukemia, and thrombosis-free survival and, to some extent, overall survival too. Could the authors postulate a reason for this, specifically, are these bone fide ET patients given their apparent more “benign course?

Finally, the clinical course of the Mayo Clinic TN patients was not as favorable as those from Florence (TN patients fared better only regarding overall survival, leukemia-free survival, and arterial thrombosis-free survival). Do the authors have an explanation for this or are the findings in both cohorts “close enough” to represent an accurate natural TN ET disease course?
